# Affective Compatibility between Stimuli and Response Goals: A Primer for a New Implicit Measure of Attitudes

**DOI:** 10.1371/journal.pone.0079210

**Published:** 2013-11-14

**Authors:** Andreas B. Eder, Klaus Rothermund, Jan De Houwer

**Affiliations:** 1 Department of Psychology, University of Würzburg, Würzburg, Germany; 2 Department of Psychology, University of Jena, Jena, Germany; 3 Department of Experimental Clinical and Health Psychology, Ghent University, Ghent, Belgium; Centre national de la recherche scientifique, France

## Abstract

We examined whether a voluntary response becomes associated with the (affective) meaning of intended response effects. Four experiments revealed that coupling a keypress with positive or negative consequences produces affective compatibility effects when the keypress has to be executed in response to positively or negatively evaluated stimulus categories. In Experiment 1, positive words were evaluated faster with a keypress that turned the words ON (versus OFF), whereas negative words were evaluated faster with a keypress that turned the words OFF (versus ON). Experiment 2 showed that this compatibility effect is reversed if an aversive tone is turned ON and OFF with keypresses. Experiment 3 revealed that keypresses acquire an affective meaning even when the association between the responses and their effects is variable and intentionally reconfigured before each trial. Experiment 4 used affective response effects to assess implicit in-group favoritism, showing that the measure is sensitive to the valence of categories and not to the valence of exemplars. Results support the hypothesis that behavioral reactions become associated with the affective meaning of the intended response goal, which has important implications for the understanding and construction of implicit attitude measures.

## Introduction

Social psychology has benefitted from the use of reaction time tasks that allow one to make inferences about attitudes, stereotypes, and other types of social knowledge. In so-called implicit attitude measures, for instance, participants typically categorize attitude stimuli with two response keys; attitudes are then inferred from differences in response times on different types of trials. When using these measures, however, it is important not only to understand *what* is measured but also *how* the measurement outcome was produced [1], [2]. In this article, we discuss the role of action goals in implicit attitude tasks, and how an affective correspondence relation between attitudinal objects and intended action effects can be used to draw inferences about attitudes.

### Affective Stimulus-Response Compatibility Tasks

In cognitive psychology, the observation that some reactions are emitted more easily than others in response to specific stimuli is captured by the notion of stimulus-response compatibility (SRC): A response is selected more quickly when a relevant or irrelevant stimulus feature is somehow related or similar to the correct response than when both elements differ. For instance, a left key is pressed more quickly in response to a stimulus positioned at the left side of the screen than to a stimulus presented at the right side, whereas the reverse is observed with a right key press. Thus, responses are faster when there is spatial correspondence between the stimulus and the correct response and slower when there is correspondence between the stimulus and the alternative response, even when the stimulus position is completely irrelevant to the task at hand (the so-called Simon effect).

Importantly, a similarity or correspondence relation between stimuli and responses is not restricted to physical response dimensions like spatial orientation; instead, any conceptual, structural, or perceptual similarity relation between stimuli and responses can produce an SRC effect [3]. A correspondence relation that is of special importance to social psychological research is manipulated in affective SRC paradigms (for an overview see [4], [5]). In these tasks, both stimuli and responses have an affective meaning or implication, and task instructions establish either affectively compatible (same valence) or affectively incompatible (different valence) S-R combinations. In affective variants of the Simon task, for instance, affective responses (e.g., the pronunciation of GOOD and BAD) are emitted on the basis of an affectively neutral feature (e.g., the grammatical category of words) of positive and negative stimuli. It is typically observed that responses are faster when the affective valence of the response is congruent with the affective valence of the stimulus relative to when they are incongruent [6]. Like other types of compatibility effects, affective SRC effects are predicted by cognitive-coding accounts that assume that both stimuli and responses are cognitively represented by means of codes and that selection of a response is facilitated by a code match and misled by a code mismatch [7].

Several authors (e.g., [4], [5], [8]) have suggested that affective SRC also operates in popular attitude measurement tasks like the affective priming paradigm [9], the extrinsic affective Simon task (EAST; [10]), and the Implicit Association Test (IAT; [11]). In the affective priming paradigm, for instance, participants respond to positive and negative targets more quickly and/or more accurately when the targets are preceded by affectively compatible primes (e.g., nice-sun) than when they are preceded by affectively incompatible primes (e.g., nasty-sun). Several studies have provided evidence that much of this performance difference is caused by an automatic activation of the responses as the result of the presence of the prime ([12]–[15]). For instance, Eder and colleagues [16] recorded the lateralized readiness-potential (LRP) in the human brain as an online-index of activation of the left and right response hand. Results showed that the incorrect response hand is activated more strongly in affectively incompatible trials than in affectively compatible trials, suggesting a response conflict in incompatible priming trials. Notably, this response conflict occurs even though the responses themselves are relatively neutral, such as pressing a left or right key. To account for these results in terms of affective stimulus-response conflicts, it has been suggested that neutral responses can become endowed with affective meaning as the result of task instructions that assign these responses to positive and negative words. These instructions are assumed to create short-term links between responses and the concepts “positive” and “negative” [10]. Positive and negative primes then activate responses based on the long-term associations between the stimuli and positive and negative concepts and the short-term associations between the latter concepts and the response representations. This allows affective stimuli to prime intrinsically neutral behavioral responses that are used to express evaluative decisions.

Similar ideas were put forward to explain IAT effects. In the IAT, two binary categorization tasks are intermixed within a block (attribute and target categorization), with two concepts being mapped onto each of two response keys. Selection of the response is typically faster when the concepts that are assigned to one key are somehow similar or associated in memory than when the concepts are unrelated or dissimilar. In such a situation, stimulus-response conflicts can arise because intrinsically neutral responses (i.e., pressing a left or right key) become temporarily associated with the concepts that are assigned to the responses in the task instructions [10], [17]. When participants are instructed to give a certain response (e.g., press a left key) to exemplars of an attribute concept (e.g., positive words), a short-term association is created between the representation of the response and the representation of the assigned concept. This temporary meaning of a response is then maintained even when the meaning is not relevant for the target categorization task that is also part of the combined block (e.g., when participants categorize names of flowers and insects rather than positive or negative words). Configured in this way, stimuli automatically prime the response that is associated with a similar (congruent) meaning (e.g., flower names activate the response assigned to positive words), which is the correct response in the block with compatible mappings (e.g., flower names assigned to the same key as positive words) but the incorrect response in the block with incompatible mappings (e.g., flower names assigned to the same key as negative words).

### Stimulus-based vs. Intention-based Response Coding

When participants sort different stimuli in reaction time tasks by abstract features, such as gender, race, or evaluative meanings, they construct different stimulus categories on the basis of these features that enable a meaningful stimulus sorting according to the task instructions [18]. However, besides categorizing the stimuli with respect to one or more attended features, the participants also have to indicate the result of the stimulus categorization by pressing one of several response keys according to a predefined S-R mapping. In order to implement these mapping rules, the responses must be defined in one or the other way. That means, not only the stimuli but also the responses have features, which are mentally represented in terms of response categories. A match between stimulus and response features and their respective mental representations facilitates the selection of the correct response relative to a feature mismatch, producing an SRC effect [3].

Research into SRC effects has demonstrated that people switch flexibly between a stimulus-based and an intention-based coding of the responses depending on which feature is emphasized in the task instructions. For instance, Hommel [19] instructed participants to respond to tones, presented to the left or the right ear, with a left and a right response key that flashed a light opposite to the key location (i.e., the left key turned on a light on the right side, and the right key turned on a light on the left side). When participants were instructed to press the keys in response to the auditory tones (i.e., stimulus-based coding), a standard spatial Simon effect was obtained (i.e., responses were faster when the irrelevant location of the tone corresponded with the position of the response key). In contrast, when instructions were to turn the lights on with the keys (i.e., intention-based coding), the Simon effect was reversed (i.e., responses were now faster when the tone location was compatible with the spatial position of the intended light flash but incompatible with the location of the key). Obviously, features of a response were weighed differently depending on how crucial they were for realizing the task goal (for related evidence see [20], [21]).

Similar observations were made in affective tasks in which participants responded to positive and negative stimuli with a lever pull and push. In one study [22] instructions described these lever movements either as motions towards and away from the body (self-related movement coding) or as motions towards and away from the stimulus (object-related movement coding). Both instructions imply an intention-based coding because identical movements were executed in the pursuit of different goals (e.g., the goal to push the lever “away from the body” versus the goal to push the lever “towards the stimulus”). Results showed that the nature of the instructed movement goal did matter and thus that the observed effects depended on the intention-based coding of the responses. More specifically, with the self as instructed point of reference, the lever was pulled faster in response to positive words than to negative words and pushed faster in response to negative stimuli than to positive stimuli, while the opposite was true with the stimulus as active point of reference. Furthermore, Eder and Rothermund [23] observed that pushing a lever is facilitated by positive stimuli when referred to as a movement in an “upward” direction (positive movement coding) but delayed when referred to as a movement “away” from the body (negative movement coding), while pulling the lever is facilitated by negative stimuli when instructed as “downward” movement (negative movement coding) but delayed when instructed as a movement “towards” the body (positive movement coding). Thus, the same movement was executed with different ease in response to positive and negative stimuli depending on the response goal that was emphasized in the task instructions.

Response features that are crucial for realizing the task goal thus are weighed more for action coding than features that are less central for solving the task at hand. This view fits with the intentional-feature-weighting hypothesis proposed by Hommel and colleagues ( [24], see also [25]). According to this hypothesis, a simple S-R instruction (e.g., “When you see a positive word on the screen, then move the lever towards your body”) creates a mental link between the stimulus and the instructed response goal by activating and linking their corresponding mental representations (i.e., “positive” and “towards the body” in the example). The specific motor codes that are needed to perform the instructed response are then primarily accessed via the mental representation of the instructed response goal (i.e., “towards the body”). Thus, while the mental representation of an action contains both instructed and noninstructed features, the action is primarily accessed by the codes that represent the instructed response goal [26].

### The Purpose of the Present Study

As is clear from the previous sections, SRC models of implicit attitude measures assume that responses become associated with an affective meaning as the result of being assigned to positive or negative stimuli. It is still unclear, however, why a task to respond to positive and negative concepts by pressing one of two keys endows those responses with a temporary affective meaning. At least two explanations are possible: First, the affective concept is instructed as an antecedent condition that specifies when to perform what action (e.g., “press the left key when a positive stimulus appears on the screen”). Second, the affective concept is introduced as a reason or as a goal for a specific response (e.g., “press the left key in order to signal the presence of a positive stimulus”). Note that responses can derive a meaning from both task components. For instance, a left response assigned to a positive concept can have a positive meaning because the response is emitted when a positive stimulus is present (stimulus-based response coding) or because of the behavioral intention to signal the presence of a positive stimulus to the experimenter (intention-based response coding). Accordingly, it is not clear whether the meaning of a response is based on the concept that was specified for the antecedent part of a response or by the concept that was specified for the goal part of the response (or by both).

The present studies disentangled these task components by introducing affective response effects that were not tied to evaluations of affective stimuli. Participants were to evaluate affective words as quickly and as accurately as possible with a left and a right response key. In addition to this clear S-R mapping, the key responses were furnished with functions that were irrelevant for the task at hand (i.e., word evaluation). For instance, in one experiment a left key turned words ON and a right key turned words OFF. Participants were instructed to produce these effects depending on whether the presented word is positive or negative. In line with the intentional-coding hypothesis, we expected that the responses become associated with the intended effect, creating affectively congruent and incongruent combinations between words and responses.

Examining such effects is important for several reasons. First, it improves our understanding of how neutral responses can become endowed with an affective meaning, and thus how affective SRC effects can arise in implicit attitude measures. Second, showing such effects would provide more evidence for SRC models of implicit attitude measures that propose that behavioral responses become associated with a temporary affective valence that interacts with affective meanings of stimuli (e.g., [17]). This is important because at present the evidence for affective SRC models of measures with intrinsically neutral responses is limited (if not absent). Finally, the present research paves the way for a new class of implicit attitudes measures that are based on affective SRC in which response valence is induced by the response effects. Using such a task procedure helps to overcome problematic aspects of established task procedures such as an intermixing of an additional evaluation or attribute task into the procedure (e.g., problems of recoding, [27]–[28], and task-switch costs; [29]–[30]). Using action goals (or action effects) to endow neutral responses with a positive or negative valence thus may allow us to devise implicit attitude measures that are briefer, less complicated, and more valid.

## Experiment 1

Experiment 1 provides a first test of the hypothesis that intrinsically neutral key responses derive a meaning from intended response effects. Participants were instructed to turn affective words ON and OFF with a left and a right response key. To visualize this effect, the key that turned a word ON increased the visual contrast between the word and the background color of the computer screen, whereas the key that turned words OFF decreased the contrast. It was hypothesized that turning the word ON has a positive implication, while turning the word OFF has a negative implication [31], [32]. Hence, instructions to turn positive words ON and negative words OFF were expected to be congruent, whereas instructions to turn negative words ON and positive words OFF were incongruent. As a result, performance should be better with the first than with the second set of instructions.

### Method

#### Participants

Forty students (27 women) volunteered for the experiment in fulfillment of course requirement or for payment. Participants were between 18 and 33 years old (*M* = 22.3 years). The research is in compliance with the American Psychological Association’s (APA) Ethical Principles of Psychologists and Code of Conduct. Participants in Experiment 1, 3, and 4 gave a verbal informed consent to participate in the study, and an information letter was handed out to them prior to the experimentation that informed them about their rights. A verbal consent was obtained for these experiments, because they involved simple and standard procedures without any risks (word categorizations with keypresses on a computer keyboard). Consent was acknowledged by a research assistant but not stored in written form to secure anonymity of all participants. This procedure (of verbal consent) was generally approved by the Ethics Committee of the Faculty of Social and Behavioral Sciences of the University of Jena (Germany) and by a research grant from the German Research Foundation awarded to the first author (DFG Ed201/2).

#### Material

Participants evaluated 24 positive and 24 negative adjectives that were selected from a standardized word pool according to their valence norms [33] (see Stimuli S1). The subsets of positive and negative words were matched in length (range: 4–9 letters) and frequency of usage (with both *F*s<1). An additional 6 positive and 6 negative adjectives were used for task practice. The words were presented in lower case letters at the centre of the computer screen.

#### Procedure

Participants were to evaluate affective words as fast and correctly as possible with a press of a left key (‘d’) and a right key (‘l’) on a standard keyboard. One response key turned the word ON; the other key turned the word OFF. Assignment of the effects to the left and right response keys was counterbalanced across participants. No cover story was presented for why the keys produce these effects.

To visualize the response effect, the contrast between the word color and the background color of the screen was regulated with the response keys. For half of the sample, the background of the screen was black; for the other half, the background color was white. At the start of a trial, a word always appeared in grey color. When the background was set to black, turning a word ON changed its color to white, whereas turning the word OFF made it black. When the background was set to white, in contrast, turning the word ON made it black, whereas turning the word OFF made it white. With changes of the background color, we thus disentangled the goal of turning the word ON and OFF from experiences of dark and light letter colors. Task displays with a black and a white background are illustrated in Display S1.

In one half of the experimental blocks, participants were instructed to turn the word ON when it is positive and OFF when it is negative (congruent mapping). In the other half of the blocks, the assignment of the responses and their effects to the evaluative categories was reversed (incongruent mapping). The order of the blocks with a congruent and incongruent response assignment was counterbalanced across participants.

In addition to the word evaluation task, participants were occasionally to respond to the words ‘AN’ (on) and ‘AUS’ (off) with a press of the designated key (so-called label trials; cf. [23]). These ‘label trials’ were randomly intermixed to maintain the instructed response coding throughout the entire experiment and to prevent participants from recoding the meaning of the visual response effects. The words AN and AUS were written in uppercase letters to make them more distinctive from the to-be-evaluated adjectives. Label trials comprised only one fifth of the block trials to ensure that the word evaluation is the dominant task.

An experimental trial started with a brief presentation (200 ms) of a fixation sign (asterisk) in the middle of the screen. Following an additional interval of 100 ms, a word was presented in grey color until a key was pressed. After a delay of 50 ms, and depending on the color condition, the word gradually turned black or white for 500 ms. The response effect appeared after every response, with one response key turning the word on and the other response key turning the word off irrespective of the valence of the target. However, an error feedback was given on an incorrect button press and on slow responses (latency greater than two seconds) for 500 ms at the end of a trial. The next trial started after 1 sec.

The experiment consisted of 240 experimental trials, subdivided into eight blocks with 30 trials each. In each block, 12 positive and 12 negative adjectives were presented for evaluation; in the remaining 6 trials, the words ‘AN’ (ON) and ‘AUS’ (OFF) were presented three times (label trials). In two consecutive blocks, all 48 adjectives were presented in a randomized order. The assignment of the keys to the evaluative categories changed after the first four blocks, and participants completed 18 practice trials (12 evaluation trials and 6 label trials) before the start of the first and fifth experimental block to become familiar with the (new) mapping rules.

After the reaction time task, participants were asked to rate the words LINKS (LEFT), RECHTS (RIGHT), AN (ON), AUS (OFF), WEISS (WHITE), SCHWARZ (BLACK), HELL (BRIGHT), and DUNKEL (DARK) on a scale ranging from −4 (very negative) to +4 (very positive) in random order. At the end of the session, participants were asked for biographical data, thanked, and dismissed.

### Results

Trials with incorrect responses (5.9% of the trials) were removed from the reaction time analyses. In addition, individual Tukey [34] outlier thresholds removed 4.9% of the response times. Response times and error rates are shown in Table S1. For all analyses, the significance criterion was set to *p<*.05 (two-tailed). Confidence intervals of standardized effect sizes (Cohen’s d, partial eta-square) were computed with the package “MBESS” for the statistical software R [35]. The assignment of the response goal to the left and right response keys had no effect on the results; therefore, data were collapsed across both groups in the subsequent analyses.

**Figure 1:**
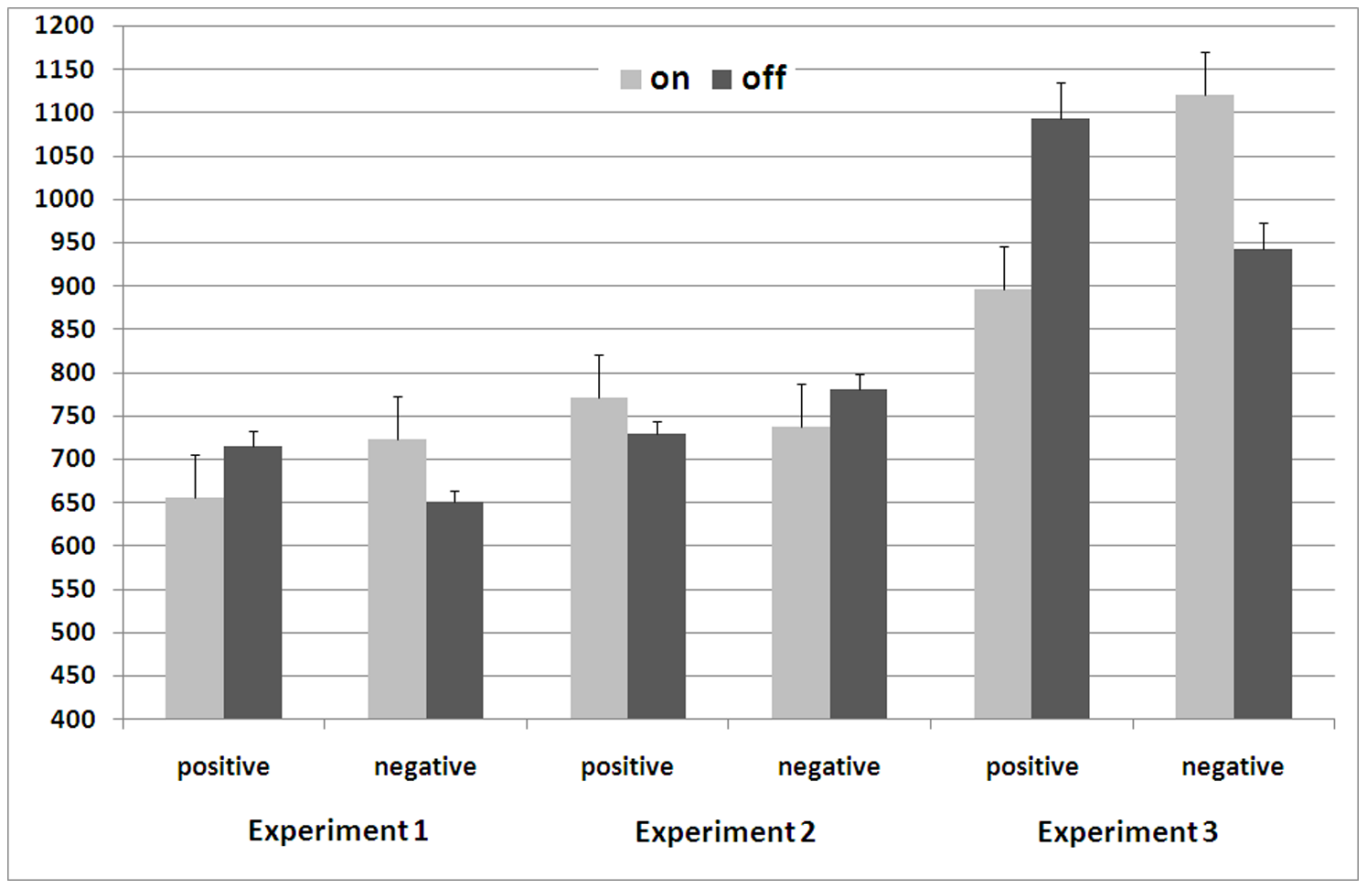
Reaction times (in ms) as a function of stimulus valence and response goals in Experiment 1 to 3. Error bars show the standard error.

A mixed-model ANOVA of the reaction times with stimulus valence and response goal (on vs. off) as within-subject factors and mapping order and background color as between-subjects factors yielded the expected interaction between stimulus valence and response goal (*d* = 0.89, 95% CI [0.52, 1.25]), *F*(1, 36) = 36.01, *p*<.001, which was not qualified by the background color (*F*<1). As illustrated in Figure 1, keys that turned positive words on and negative words off were pressed faster (*M* = 654 ms, *SE* = 14.4) than keys that turned negative words on and positive words off (*M* = 719 ms, *SE* = 17.7). As indicated by a significant three-way interaction with mapping order, the congruency effect was enhanced when participants received an incongruent key mapping first (Δ*M* = 88 ms, *d* = 1.12, 95% CI [0.55, 1.67]) relative to when congruent blocks were completed first (Δ*M* = 43 ms, *d* = 0.70, 95% CI [0.20, 1.18]), *F*(1, 36) = 4.18, *p*<.05, η_p_^2^ = .104, 95% CI [0,.290], but the effect was significant from zero with both orders, *t*(19) = 5.01, *p*<.001, and *t*(19) = 3.11, *p*<.05. No other effects were significant (all *F*s<3.10).

In an analogous analysis of the error rates, the interaction between stimulus valence and response goal was not significant (*F*<1). Participants made fewer errors when the word was positive, *F*(1, 36) = 6.38, *p*<.05, η_p_^2^ = .151, 95% CI [.004,.341]. Furthermore, error rates differed with the background color when congruent blocks came first but not when the incongruent blocks were first, *F*(1, 36) = 4.89, *p*<.05, η_p_^2^ = .120, 95% CI [0,.308]. All other effects were not significant (all *F*s<3.77).

#### Label rating

Participants judged ON more positively (*M* = 2.1) than OFF (*M* = −2.0) (*d* = 1.49, 95% CI [1.03, 1.94]), *t*(39) = 9.45, *p*<.001. In addition, evaluative ratings were different for WHITE (*M* = 1.8) and BLACK (*M* = −1.1) (*d* = 1.09, 95% CI [0.69, 1.48]), *t*(39) = 6.91, *p*<.001, and for BRIGHT (*M* = 3.0) and DARK (*M* = −2.0) (*d* = 2.01, 95% CI [1.46, 2.55]), *t*(39) = 12.72, *p*<.001. LEFT (*M* = −0.05) and RIGHT (*M* = −0.08) were not rated differently (*t*<1).

### Discussion

Participants turned positive words faster ON than OFF and negative words faster OFF than ON. This result confirms that the valence of the responses was influenced by the behavioral intention to turn words ON and OFF. Furthermore, the congruency effect was enhanced when participants received an incongruent key mapping first. This compatibility-order effect may be due to task practice. With generally slower responses in the first task blocks (*M* = 697 ms) than in the second task blocks (*M* = 675 ms), task practice enhanced the reaction time difference between both conditions when the incongruent blocks came first but decreased the reaction time difference when the congruent blocks came first. Given that a congruency effect was obtained with both mapping-orders, however, it is also clear that the effect cannot be attributed to task practice alone.

## Experiment 2

According to the intentional-coding hypothesis, meanings attached to responses should depend on the goals they are in service for; thus, the affective implication of the intended effect should determine the affective meaning of a response. Experiment 2 tested this hypothesis with key responses that turned a loud noise ON and OFF. Given that noise is an aversive consequence, we hypothesized that the response that turns the noise ON acquires a negative valence, whereas the response that turns the noise OFF acquires a positive valence [36]. As a result of this affective coding, the key turning the sound OFF should be pressed faster in response to positive words (relative to negative words) and the key turning the noise ON should be pressed faster in response to negative words (relative to positive words). In short, we expected a response facilitation effect that is exactly opposite to the effect that was observed in the previous experiment in which responses served to switch words ON and OFF.

### Method

#### Participants

Thirty-nine students (27 women) took part in the experiment for course credit or for payment. Participants were between 19 and 45 years of age (*M* = 24.0 years). Participants in Experiment 2 signed a written informed consent document that disclosed their rights and the experimental treatment. The experiment was approved by a local ethics committee of the University of Jena (FSV 10/01).

#### Stimuli, design, and procedure

Stimuli, design, and procedure were the same as in Experiment 1 except for the following changes: (1) Prior to the experiment, an informed consent was obtained from all subjects. Participants were informed that the experiment investigates response performance under stress and that they will occasionally hear loud noise that can be turned on and off with two keys: one key turns the noise ON, whereas another key turns the noise OFF. The key assignment was counterbalanced across participants.

(2) The noise was a highly aversive sound that was used in earlier research for punishment [37]. The sound was presented binaurally via headphones, and the noise level was individually adjusted to the participant’s threshold prior to the experiment. The sound intensity was adjusted by using the volume control of the operating computer system, and the volume setting was then constant throughout the experiment. During the experiment, the noise was turned ON when participants responded to the word “AN” (on) with a respective key press. The noise signal was then continually replayed until a response was made to the word “AUS” (off). Thus, keys were only effective in turning the noise ON and OFF when the words AN and AUS appeared (i.e., in the label trials) but not when adjectives were categorized (i.e., in the evaluation trials). By extending the time period of the presence and absence of the noise, we intended to increase the motivational relevance of turning the noise ON and OFF (cf. [38]).

(3) A loudspeaker icon appeared after each key press that was crossed out when the key turning the noise OFF was pressed. The icon was black when the response was correct and red when incorrect. The visual icons were presented to support a response coding in terms of the sound effects during the evaluation trials, in which a button press had no obvious effect on the presentation of the sound (see the task procedure above). The visual display presented for this task is illustrated in Display S2.

(4) To prevent strategic avoidance of turning the noise ON, a monetary penalty was introduced for omitted and incorrect responses. Participants started with a credit of 400 Eurocents. From this credit, 5 Cents were deduced for an incorrect response in the evaluation trials, and 10 Cents were deduced for an incorrect or omitted response in the label trials. In erroneous trials, a message appeared for 500 ms that specified the penalized error (e.g., “INCORRECT: -5 Cents”). Participants were informed about the credit balance after each experimental block.

(5) To familiarize the participants with the key functions, the experiment started with a practice block (12 trials) that exclusively presented the words “AN” and “AUS” in random order. This practice was continued until the correct key was pressed in every trial. Then, a practice block (16 evaluation trials and 4 label trials) and a trial block (48 evaluation trials and 12 label trials) were presented for each mapping condition.

(6) After completion of the reaction time task, participants were asked to rate LINKS (LEFT), RECHTS (RIGHT), AN (ON), AUS (OFF), LEISE (QUIET) and LAUT (LOUD) in random order.

### Results

Trials with incorrect responses (6.0% of all trials) were dismissed from reaction time analyses. In addition, individual Tukey [34] outlier thresholds removed 4.5% of all response times. Response times and error rates are shown in Table S2. The assignment of the response goal to the left and right response keys had no effect on the results; therefore, data were collapsed across both groups in the subsequent analyses.

A mixed ANOVA of the reaction times with stimulus valence and response goal (on vs. off) as within-subject factors and mapping order as between-subjects factor yielded a significant interaction between stimulus valence and response goal (*d* = 0.61, 95% CI [0.26, 0.95]), *F*(1, 37) = 14.03, *p*<.001. As shown in Figure 1, participants responded faster to positive words with the key that turned the noise OFF and to negative words with the key that turned the noise ON (*M* = 733 ms, *SE* = 14.8) than with the reverse key assignment (*M* = 776 ms, *SE* = 16.6). No other effects were significant (with all *F*s<2.44).

In an analogous ANOVA of the error rates, only the three-way interaction between stimulus valence, response goal, and mapping order reached significance, *F*(1, 37) = 10.72, *p*<.05, η_p_^2^ = .225, 95% CI [.033,.423]. When the incongruent block came first, a congruency effect was obtained; in contrast, an opposite trend was observed when participants started with the congruent key mapping. All other effects were not significant (with all *F*s<2.42).

#### Label rating

LEFT (*M* = 0.05) and RIGHT (*M* = 0.13) were not evaluated differently (*t*<1). QUIET (*M* = 1.9) was evaluated more positively than LOUD (*M* = −1.2) (*d* = 0.89, 95% CI [0.51, 1.26]), *t*(38) = 5.58, *p*<.001. Furthermore, and in line with the motivational manipulation, ON was judged more negatively (*M* = −0.1) than OFF (*M* = 0.3), even though this difference was not significant (*t*<1). Ratings of ON and OFF in this experiment were however opposite to those obtained for Experiment 1 (*d* = 1.13, 95% CI [0.65, 1.60]), *t*(77) =  −5.03, *p*<.001. Thus, participants judged the affective meaning of the words ON and OFF differently, depending on whether a light (word) or a noise was turned OFF and ON in the experiment.

### Discussion

In this experiment, a response that turned a noise signal OFF was emitted faster in response to positive words than to negative words, while the opposite was true with responses that turned the noise ON. This pattern of response facilitation, and the explicit ratings of ON and OFF, are in line with the hypothesis that turning an aversive event ON endows the associated action with a negative meaning, whereas turning the noise OFF endows the response with a positive meaning. Notably, the response-facilitation effect is exactly opposite to the facilitation pattern observed in Experiment 1, in which the response keys produced relatively neutral outcomes (i.e., turning words ON and OFF). Obviously, then, the affective implication of ON and OFF depends on what kind of event is turned on and off, confirming that the affective coding of a response is sensitive to the motivational implications of the produced effect.

## Experiment 3

The experiments described so far support the hypothesis that responses derive a meaning from intended response goals. In these experiments, however, participants were also instructed to respond to the words ON and OFF in label trials that were occasionally intermixed to disambiguate the meaning of the visual response effects. Accordingly, it is not clear whether the responses derived a meaning from the relation with the produced effects or from the responses to the words that described these effects in the intermixed label trials (or both). The aim of Experiment 3 was to examine pure effects of response goals on intentional response-coding.

Experiment 3 was similar to Experiment 1 except for the omission of label trials. Participants were to turn words ON and OFF by pressing a left and right response key. In order to increase the salience and importance of the action effects for the representation of the responses and to prevent a (neutral) recoding of the responses during the experiment, we randomly changed the assignment of keys to the positive and negative consequences between trials: Which of the two response keys turned the word ON or OFF was cued before each trial. (This task procedure was inspired by an approach-avoidance task in which a single response key is pressed to steer a manikin towards and away from an affective object depending on the manikin’s start position ([39], Exp. 4). Accordingly, the assignment of the response effects to the response keys was not fixed, and participants had to intentionally reconfigure the relation between the responses and their effects before each trial.

### Method

#### Participants

Thirty-four students (17 women) participated for course credit or for payment. Participants were between 19 and 27 years of age (*M* = 21.8 years). The data of one participant was not analyzed because he responded incorrectly in 48% of the trials.

#### Stimuli, design, and procedure

**Figure 2:**
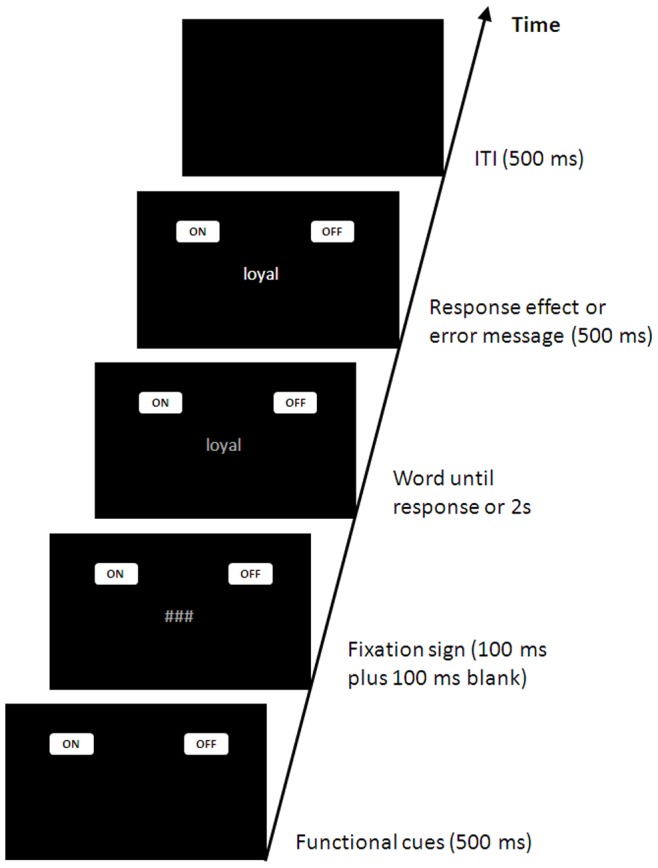
Sequence of events in a trial of Experiment 3 in which a word is turned on with a left button press. Note that the position of the functional cues ON (AN) and OFF (AUS) could change from trial to trial.

Stimuli, design, and procedure were the same as in Experiment 1 except for the following changes: (1) Participants worked through 192 evaluation trials; no label trials were presented. (2) Participants evaluated grey words presented on a black screen by pressing keys that turned the word ON (effecting a color change to white) and OFF (effecting a change of the word color to black) (for an illustration of the visual displays see Display S3). The function of a key in a trial was cued by the horizontal position of the words AN (OFF) and AUS (OFF) that appeared in white boxes (100 pixels wide and 60 pixels high) at the upper left and right corners of the screen: When ON appeared at the left corner and OFF at the right corner, a press of the left key turned the word on and a press of the right key turned the word on. When the response cues appeared at the opposite locations, the assignment of the key functions was reversed as well. Participants were instructed to look at the position of the words ON and OFF at the start of a trial, and to press the response key that produces the corresponding effect according to the task instructions. (3) In half of the trials, ON appeared at the left corner and OFF at the right corner; in the remaining trials, the location of the response cues was reversed. The assignment of the cue location was random with the restriction that an assignment rule was not repeated in more than three trials in a row. (4) Figure 2 shows the sequence of events in an experimental trial. The response cues were presented at the corners 500 ms before the onset of the to-be-evaluated word and they remained there visible until the end of a trial. A response effect appeared only after a correct button press; in the case of an incorrect or omitted response, an error message that provided information about the type of error, appeared for the same time period as the response effect would have been (i.e., 500 ms). On all trials, the ITI was 500 ms.

### Results

Participants pressed the incorrect key in 13.3% of the trials. These trials were dropped from reaction time analyses. In addition, individual Tukey [34] outlier thresholds removed 2.5% of the reaction times. Response times and error rates are reported in Table S3.

A mixed ANOVA of the reaction times with stimulus valence and response goal (on vs. off) as within-subject factors and mapping order as between-subjects factor yielded a significant main effect of stimulus valence, *F*(1, 31) = 19.09, *p*<.001, η_p_^2^ = .381, 95% CI [.118,.568], and a significant interaction between stimulus valence and response goal (*d* = 0.96, 95% CI [0.54, 1.37]), *F*(1, 31) = 38.01, *p*<.001. As Figure 1 shows, responses were faster when the intended goal was congruent with the stimulus valence (*M* = 917 ms, *SE* = 28.6) than when the goal was incongruent (*M* = 1107 ms, *SE* = 39.2). The size of the congruency effect was additionally influenced by the order of the mapping instructions, *F*(1, 31) = 8.11, *p*<.05, η_p_^2^ = .207, 95% CI [.016,.422]. Follow-up tests revealed an enhanced congruency effect when participants worked through the incongruent block first (Δ*M* = 280 ms, *d* = 1.67, 95% CI [0.89, 2.43]) compared to when they received congruent mapping rules first (Δ*M* = 102 ms; *d = *0.55, 95% CI [0.03, 1.05]). However, the congruency effect was significant in both conditions, *t*(15) = 6.69, *p*<.001, and *t*(16) = 2.27, *p*<.05. All other effects were not significant (all *F*s<1).

An analogous analysis of the error rates yielded a main effect of stimulus valence, *F*(1, 31) = 5.33, *p*<.05, η_p_^2^ = .147, 95% CI [0,.363], and significant interaction between response goal and mapping order, *F*(1, 31) = 5.70, *p*<.05, η_p_^2^ = .155, 95% CI [.001,.372]. Most important, the interaction between stimulus valence and response goal was significant, *F*(1, 31) = 20.66, *p*<.001, confirming a congruency effect (ΔM = 7.8%, *d* = 0.80, 95% CI [0.40, 1.19]). All other effects were not significant, including the three-way interaction (with all *F*s<1).

#### Label rating

Evaluative ratings of ON and OFF were in line with those obtained in Experiment 1. ON was rated more positively (*M* = 2.2) than OFF (*M* = −1.9) (*d* = 1.40, 95% CI [0.91, 1.88]), *t*(32) = 8.04, *p*<.001. BRIGHT (*M* = 2.5) was evaluated more favorably than DARK (*M* = −1.0) (*d* = 0.99, 95% CI [0.57, 1.40]), *t*(32) = 5.68, *p*<.001. WHITE (*M* = 1.8) was judged more positively than BLACK (*M* = −0.3) (*d* = 0.69, 95% CI [0.31, 1.07]), *t*(32) = 3.94, *p*<.001. Evaluative ratings of LEFT (*M* = 0.1) and RIGHT (*M* = 0.3) were not different (*t*<1).

### Discussion

The results of Experiment 3 closely replicate those of Experiment 1 despite the removal of intermixed label trials. Again, participants found it easier to turn positive words ON and negative words OFF than vice versa. This finding shows that an intention to turn words ON and OFF with keypresses is sufficient to endow the response keys with an affective meaning.

## Experiment 4

Experiment 4 used the task procedure for a measurement of in-group favoritism, which is a tendency “to evaluative one’s own membership group (the in-group) or its members more favorably than a nonmembership group (the out-group) or its members” ([40], p. 576). German students who participated in this experiment were instructed to discriminate the nationality of German and foreign person names by pressing one of two keys. One response key generated a picture with a thumb-up gesture on the computer screen that signaled TOP; the other key produced a thumb-down gesture on the screen that signaled FLOP. TOP and FLOP were used to disambiguate the meaning of the hand gestures. Participants were instructed to produce the thumb-down gesture (FLOP) and the thumb-raised gesture (TOP) with a corresponding key press depending on whether a name of a German person or a foreign person is displayed on the screen. In line with research on an implicit in-group bias (e.g., [41]), we expected faster and more accurate responses in trial blocks in which the assignment of the hand gestures (i.e., response keys) was compatible with an ingroup favoritism (i.e., German name-TOP, foreign name-FLOP) compared to when it is not (i.e., German name-FLOP, foreign name-TOP).

In addition to the task-relevant compatibility relation between the nationality and the hand gestures, we also varied the compatibility relation between the valence of the group exemplars and hand gestures that was irrelevant for the task at hand. Half of the German and foreign persons were liked by the participants (e.g., Friedrich Schiller; Dalai Lama), whereas the other German and foreign persons were disliked (e.g., Adolf Eichmann; Silvio Berlusconi). In line with previous research on affective SRC effects [4], and [17], we expected a strong influence of the task-relevant compatibility relation between the national groups (German vs. foreigner) and the response goals (TOP vs. FLOP), but no systematic effect of the task-irrelevant compatibility relation with the valence of the group exemplars.

### Method

#### Participants

Thirty-one students (14 women) with an age between 21 and 42 years (*M* = 24.0 years) participated for course credit or for payment. All participants were German citizens. The data of one participant were not included in the analyses because he responded correctly in only 64% of the trials (rest of the sample: *M* = 92%, *SD* = 4.6).

#### Stimuli

Stimuli were the names of six liked German persons, six disliked German persons, six liked foreign persons, and six disliked foreign persons. The names were selected according to their evaluative norms that were obtained from a pretest-rating of 52 person names (*n* = 19). At the start of the experiment, each person was introduced to the participant by presenting his or her name (e.g., George W. Bush) together with a picture and a short biography of the person (e.g., former US president). The names and the person descriptions (translated in English) are shown in Stimuli S2.

Response goals were visualized with schematic pictures of hand gestures that showed a closed fist with an extended thumb in an upward or downward direction. TOP was visualized with a picture that showed a thumb raised up; FLOP was visualized with a 180 degrees rotated version of the picture with the thumb raised down. The pictures were 166 pixels wide and 323 pixels high and they were presented at the centre of the computer screen.

#### Procedure

The experiment was modeled after the general procedure of Experiment 3. At the start of the experiment, participants were informed that this experiment investigates how quickly people can process information about other people. Their task is to decide as quickly and as accurately as possible whether a presented name belongs to a German person or to a foreigner by pressing the keys ‘a’ and ‘l’ of the keyboard. A press of one key generated a picture with a thumbs-up gesture (TOP), and a press of the other key produced a picture with a thumbs-down gesture (FLOP) on the computer screen. No cover story was provided for why the responses produce these hand gestures. For an illustration of the task display see Display S4.

Participants were instructed to categorize the person names by lowering and raising the thumb. In one task condition, instructions were to lower the thumb if the presented name belongs to a foreign person and to raise the thumb if the name belongs to a German person (congruent mapping: German-TOP, foreign-FLOP); in the other condition, the instructions were reversed (incongruent mapping: German-FLOP, foreign-TOP). The order of the task instructions was counterbalanced across participants.

Like in Experiment 3, the function of a key was cued in each trial by the position of the words TOP and FLOP in left and right corner fields (cf. Fig. 2). In half of the trials, TOP appeared in the left field and FLOP in the right field; in the remaining trials, the placement of the functional cues was reversed. The assignment of the functional cues was random with the restriction that an assignment rule was not repeated in more than three trials in a row.

The sequence of events within a trial was identical to those of Experiment 3 (see Fig. 2). For each mapping condition, participants first worked through a practice block and then through four experimental blocks with 24 trials each. In every block, all person names were presented once in random order.

After the experimental phase, participants were asked to rate each person whose name was presented during the experiment on a scale ranging from 1 (very negative) to 9 (very positive). Furthermore, Germans and foreigners were rated collectively using the same rating scale. Ratings of TOP and FLOP were not obtained given their clear valence.

### Results

#### Liking rating

The liking scores of the postexperimental rating were in line with those obtained from the preexperimental questionnaire. A repeated-measures ANOVA with group and exemplar valence produced a main effect of exemplar valence (*d* = 3.96, 95% CI [2.88, 5.03]), *F*(1, 29) = 470.1, *p*<.001. Liked German persons (*M* = 6.9, *SD* = 1.3) were rated more favorably than disliked German persons (*M* = 2.7, *SD* = 0.9), and liked foreigners (*M* = 7.7, *SD* = 0.6) were evaluated more positively than disliked foreigners (*M* = 2.2, *SD* = 1.0). The rating difference between liked and disliked persons was more pronounced for foreign persons, as indexed by a significant interaction between exemplar valence and group (*d* = 1.14, 95% CI [0.67, 1.60]), *F*(1, 29) = 39.09, *p*<.001. Notably, Germans were not generally evaluated more favorably than foreigners, neither in the group ratings that were derived from the ratings of group exemplars (*F*<1) nor in the global rating of Germans and foreigners (*t<*1). Thus, there was no indication of an ingroup favoritism in the explicit ratings.

#### Reaction performance

Participants pressed the incorrect key in 9.1% of the trials. These trials were eliminated from the reaction time analysis. Furthermore, individual Tukey [34] outlier thresholds removed 3.0% of the reaction times. Response times and error rates are available in Table S4.

**Figure 3:**
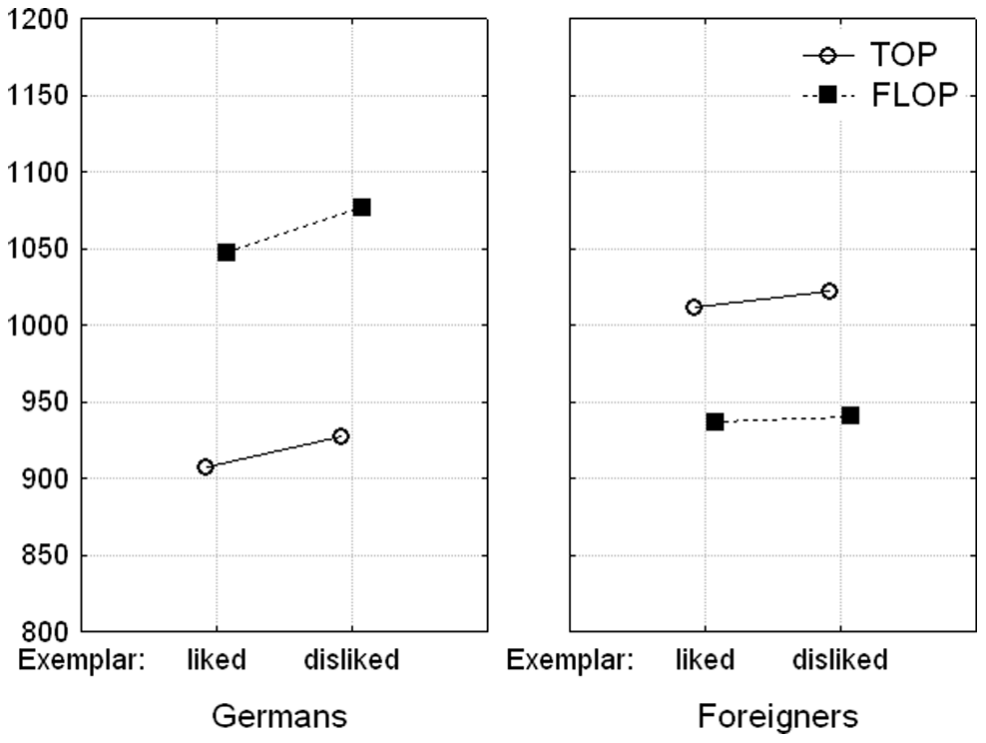
Reaction times (in ms) as a function of group (German vs. foreigner), liking of the group exemplar, and response goal (TOP vs. FLOP).

A mixed ANOVA of the reaction times with group (German vs. foreigner), exemplar valence, and response goal (TOP vs. FLOP) as within-subject factors and mapping order as between-subjects factor yielded a main effect of exemplar valence, *F*(1, 28) = 10.98, *p*<.05, η_p_^2^ = .280, 95% CI [.042,.496], and a main effect of response goal, *F*(1, 28) = 18.09, *p*<.001, η_p_^2^ = .392, 95% CI [.115,.583]. Participants responded faster to positive exemplars than to negative exemplars. Furthermore, they pressed the key that raised the thumb (TOP) faster than the key that lowered the thumb (FLOP), especially when an incongruent task instruction came first, *F*(1, 28) = 7.03, *p*<.05. Most importantly, the interaction between exemplar valence and response goal was not significant (*F*<1) but the interaction between group and response goal was (*d* = 0.72, 95% CI [0.31, 1.12]), *F*(1, 28) = 26.58, *p*<.001. As shown in Figure 3, participants responded to names of German persons faster with TOP (*M* = 917 ms, *SE* = 35.7) than with FLOP (*M* = 1062 ms, *SE* = 31.6), while the reverse was true for names of foreign persons. In these trials, participants responded faster with FLOP (*M* = 938 ms, *SE* = 36.4) than with TOP (*M* = 1017 ms, *SE* = 30.7). Notably, the congruency effect between group and response goal was not influenced by the liking of the group exemplar (*F*<1). Furthermore, the congruency effect was not systematically related to ingroup favoritism as indexed by the explicit rating scores (with *r* = .15, for the global rating, and *r* = .22, for the group rating derived from the exemplars). The size of the congruency effect was however affected by the order of the mapping instructions, *F*(1, 28) = 16.37, *p*<.001, η_p_^2^ = .369, 95% CI [.097,.565]. When the incongruent block came first, a strong congruency effect was obtained (Δ*M* = 198 ms, *d* = 1.63, 95% CI [0.81, 2.43]), *t*(13) = 6.10, *p*<.001. This effect was however reduced to an insignificant performance difference when participants worked through the congruent block first (Δ*M* = 23 ms, *d* = 0.20, *t*<1). All other effects were not significant (with all *F*s<1.89).

In an analogous analysis of the error rates, only the main effect of response goal, *F*(1, 28) = 4.80, *p*<.05, η_p_^2^ = .146, 95% CI [0,.372], and the interaction between group and response goal reached significance (*d* = 0.74, 95% CI [0.33, 1.14]), *F*(1, 28) = 16.17, *p*<.001. Errors were less frequent when participants responded to names of Germans with TOP (*M* = 4.8%, *SE* = 0.8) and to names of foreign persons with FLOP (*M* = 6.6%, *SE* = 1.1) compared to when they responded to names of German persons with FLOP (*M* = 11.3%, *SE* = 1.5) and to names of foreign persons with TOP (*M* = 9.5%, *SE* = 1.2). Like in the reaction time analysis, this congruency effect was not affected by the liking of the group exemplar (*F*<1). All other effects were not significant (all *F*s<3.53).

#### Internal consistency

Internal consistency for the implicit ingroup favoritism score was assessed by dividing the trials in four subsets by the order of their occurrence and by computing an ingroup favoritism score for each subset of trials. Cronbach’s Alpha was very high with .94 for the RT measure and .76 for the error measure. Thus, the measure is reliable.

### Discussion

The results of Experiment 4 confirm that attitudes towards the in-group are more favorable than those toward the out-group. German students who participated in our experiment responded faster to names of German persons with a symbolic hand gesture that expressed TOP compared to FLOP, while the reverse was true when a name was presented that belonged to a foreign person. This performance difference is remarkable, given that a corresponding bias was not observed in the explicit group ratings. Thus, it appears that the present task can measure implicit beliefs about social groups, that is, beliefs that people are not aware of or that they do not wish to endorse publicly. In fact, correlations between implicit and explicit ingroup favoritism scores were low, being in the same range as the ones that are typically found with more established IAT tasks [42].

Notably, the liking of the group exemplars had no effect on the response performance. Thus, the congruency effect was driven primarily by the affective properties of the category labels (German vs. foreign), which were signaled with the keypresses in line with the task instructions. This finding is in line with previous research that showed that IAT effects reflect attitudes toward the target concepts, and not attitudes toward the individual exemplars of those concepts [4], [17].

## General Discussion

When participants sort categories with keypresses in computerized attitude tasks, the responses to the category members are selected with the goal to signal the presence of a category to the computer program (or experimenter). Thus, one cannot deduce from these tasks whether a response key becomes associated with a category because of the assignment to a stimulus category or because of the intention to communicate the presence of a category member with a corresponding key press. In the present studies, this confound was removed with key functions that produced an outcome other than signaling a category member. The results consistently showed that the responses become associated with the meaning of the intended goal. This has implications for response coding, affective SRC models, and implicit attitude tasks.

### Implications for Response Coding

For the identification of correct responses in implicit measurement tasks, the responses must be defined in one or the other way. In most task situations, however, multiple frames of reference exist for action coding, and people use action frames flexibly depending on their expected utility for the task at hand [20], [43]. Consider the present Experiment 1 in which participants responded to positive and negative words with a press of the keys “d” and “l” that turned words ON and OFF. In this experiment, it was possible to select the correct response on the basis of the identity of the response keys (i.e., “d” and “l”), the location of the buttons (i.e., left key and right key), the effectors that are used to perform the response (i.e., left hand and right hand), or on the basis of the stimulus categories that were discriminated with the responses (i.e., a “positive” response key and a “negative” response key). As revealed by the results, participants identified the responses on the basis of the effects that followed contingently upon the execution of a response. This response coding in terms of the intended outcome is striking, given that it came with some performance costs in the incompatible task condition. It was however most useful for selecting a correct response in both tasks – the evaluative word decision task and the intermixed label task. A benefit in overall task performance thus may have offset a performance cost in a specific task condition, explaining why a response coding in terms of the intended outcome was maintained even when it was sometimes detrimental to performance.

**Figure 4:**
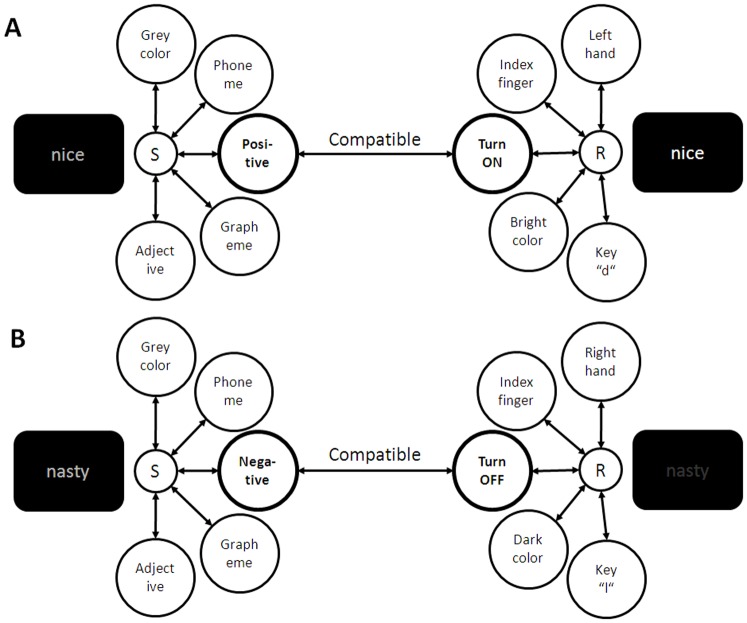
A cartoon model showing links between components of stimulus (S) and response (R) representations that are established by S-R instructions. (A) Instruction to turn a positive word on with a left key press. (B) Instruction to turn a negative word off with a right key press. Intentionally-weighted components are printed in bold.

Affective compatibility effects that are based on a response coding in terms of the intended action goals can then be explained in the following way: With the instruction to turn words on and off when a positive or negative word appears on the screen, a mental link is created between the representation of the response goal (i.e., turning the word ON and OFF) and the representation of the evaluative category (i.e., positive or negative meaning). Activation then spreads via this link from the codes representing a positive or negative word valence to the codes that represent the response goal. As shown in Figure 4, this spread of activation is additionally facilitated by a partial overlap of the codes that represent these components (i.e., an evaluative correspondence relation; see [3]). As a consequence, positive words are turned more easily on than off and negative words are turned more easily off than on with the respective button presses than vice versa.

### Implications for Affective SRC Models

Importantly, response goals may also define a response meaning in more traditional attitude tasks that do not explicitly provide key labels and/or response effects. In the IAT, for instance, an “attribute task” is used to associate neutral responses with a positive and a negative valence. When participants press a left key to positive words in an attribute IAT task, for instance, it is assumed that a short-term association is created between the representation of the response and the representation of a positive valence [4], [17]. This response meaning is then maintained also during the trials of the target-discrimination task which is part of the same block of trials (e.g., when participants must categorize flower and insect names instead of positive and negative words). As a consequence, response selection is facilitated in this task if there is a match (relative to a mismatch) between the extrinsically valenced response and the meaning of the target concepts.

According to the present theorizing, using an attribute task is an effective procedure because the instructions for this task enforce a behavior intention that constructs a corresponding response meaning. As a matter of fact, there is no structural difference between an IAT task with an intermixed attribute task and the present experiments with intermixed label trials that were used to specify a response meaning (e.g., turning words ON and OFF). (Note that an IAT measure is not only sensitive to the meaning of target concepts but also to the meaning of attribute concepts, which means that a distinction between a ‘target’ and an ‘attribute’ is not defined by task procedures but rather, by the interest of the investigator.) Given the present findings that a behavior intention suffices to attach a meaning to a response, one should consequently be able to implement a response meaning in an IAT task by task instructions alone. Consistent with this hypothesis, Banse and colleagues [44] showed that action instructions are sufficient to elicit stereotype-congruent responses in a target-discrimination task. They told young children that Santa Claus needs their help in delivering Christmas presents to a boy and a girl. Christmas presents were trucks and dolls that were assigned as quickly as possible to the boy or to the girl by pressing one of two designated keys. In a first task condition, instructions were to give dolls to the girl and trucks to the boy. In a second task condition, the assignment was reversed. Results revealed shorter responses with a stereotype-congruent assignment (i.e., truck-to-boy, doll-to-girl) than with a stereotype-incongruent assignment (i.e., truck-to-girl, doll-to-boy). Thus, the children’s intention to hand Christmas presents to boys and girls was sufficient to assign gender concepts to the response keys. Even though not discussed by the authors in this way, this finding confirms the importance of intentional response coding in the IAT and related tasks.

### Implications for Implicit Attitude Tasks

What can we learn from the present studies for a further advancement of the IAT and related attitude tasks? First, and foremost, the present studies make clear that inferences of attitudes and other types of social knowledge from performance differences in these tasks depend on how the meaning of a response is constructed on a cognitive level. Taking appropriate actions that influence this construction process in a desired direction, and taking actions that ensure that the designated response meaning is consistently maintained across participants over time, are consequently key for increasing the validity and reliability of the measurement procedure in these tasks. For instance, intermixed responses to two items that are most representative of the category distinction of interest may specify the meaning of the responses more effectively in IAT tasks than categorizations of many different individual stimuli, especially when a selection of appropriate exemplars is difficult (for evidence see [45]; but see also [46], for a diminished IAT effect with an exclusive use of category labels as items). Furthermore, a low proportion of intermixed attribute (or ‘label’) trials may suffice to specify a response meaning. In short, replacing the attribute (or target) concept task with occasionally intermixed ‘label trials’ may reduce (a) stimulus confounds of category meanings (i.e., redefinition; [47]; [48]), (b) a reframing of the response meaning (i.e., response recoding; [23]), (c) and the frequency of task switching trials, and hence a contribution of differential task-switching abilities to IAT measures [27], [28], and [49].

However, even when task switching is dramatically reduced with a low frequency of label trials, the different combination of two key mappings in separate task blocks does not preclude the possibility that participants ignore the label trials in the compatible block but not in the incompatible block (i.e., task recoding; see Rothermund & Wentura, 2004). The most rigorous way to deal with this difficulty is to dismiss label trials altogether, defining the meaning of the response keys directly in the task instructions. (Another way to prevent task recoding are trial-by-trial switches of the key assignments (IAT-RF; [50]; SB-IAT; [51]). Even though this task procedure reduces method-related variance in IAT scores effectively, the procedure is cognitively demanding and might therefore not be well-suited for a use with subject populations that have restricted cognitive capabilities (e.g., young children; see [44]). Alternatively, separating effects of associations and recoding in the IAT is possible also mathematically, with the help of multinomial modeling [52]. However, this model makes strong theoretical assumptions (e.g., unidirectionality of associations from targets to evaluative attributes but not vice versa) and may not be applicable to all kinds of IAT applications).

The Action Interference Paradigm of Banse and colleagues [44] used such a procedure successfully for a measurement of gender stereotypes, and the present studies show additional ways how even arbitrary meanings can be linked to responses. For instance, one might think of presenting the words FLOWERS and INSECTS as response-contingent effects on a computer screen and instruct participants to produce these effects with a respective button press depending on whether a presented word is positive or negative. (We have conducted this experiment (*n* = 42). Keypresses to positive words were faster when the response key produced the word FLOWERS on the screen and keypresses to negative words were faster when the response key produced the word INSECTS on the screen than vice versa (Δ*M* = 43 ms, *d* = 0.43, 95% CI [0.11, 0.74]), *t*(41) = 2.76, *p*<.01.) Alternatively, one can present pictures of flowers and insects as stimuli and instruct participants to generate a smiley and grumpy on the screen as response effects.

In sum, then, conferring evaluative meaning to responses by introducing action effects of the response keys is a very elegant way to get rid of several kinds of problems that have beset implicit attitude measures in the past. Assigning positive and negative consequences to responses in an IAT-like task allows one to eliminate effects of task-switching and recoding, which also should make the measure insensitive to effects of stimulus selection [48]. With the task procedures described in the present article, it is possible to assign even highly abstract concepts to the response keys, whilst maintaining the benefits of a single categorization task.

## Supporting Information

Table S1 Reaction times (in ms) and error rates (in percent) in Experiment 1 as a function of stimulus valence, response goal, order of the response-mapping instructions (congruent task rules first vs. incongruent task rules first), and experiment version (black background vs. white background). Standard deviation in parentheses.
(DOCX)

Table S2 Reaction times (in ms) and error rates (in percent) in Experiment 2 as a function of stimulus valence, response goal, and order of the response-mapping instructions (congruent task rules first vs. incongruent task rules first). Standard deviation in parentheses.
(DOCX)

Table S3 Reaction times (in ms) and error rates (in percent) in Experiment 3 as a function of stimulus valence, response goal, and order of the response-mapping instructions (congruent task rules first vs. incongruent task rules first). Standard deviation in parentheses.
(DOCX)

Table S4 Reaction times (in ms) and error rates (in percent) in Experiment 4 as a function of group, exemplar valence, response goal, order of the response-mapping instructions (congruent task rules first vs. incongruent task rules first). Standard deviation in parentheses.
(DOCX)

Display S1 Task displays presented in Experiment 1.
(TIF)

Display S2 Task display presented in Experiment 2.
(TIF)

Display S3 Task display presented in Experiment 3.
(TIF)

Display S4 Task display presented in Experiment 4.
(TIF)

Stimuli S1 Positive and negative words presented in Experiments 1 to 3 (English translation in brackets).
(DOCX)

Stimuli S2 Target groups and group exemplars presented in Experiment 4.
(DOCX)
