# Supplementary material for: Affective Compatibility between Stimuli and Response Goals: A Primer for a New Implicit Measure of Attitudes
Source: PLoS One. 2013 Nov 14;8(11):e79210. doi: 10.1371/journal.pone.0079210 (PMC3828340; doi:10.1371/journal.pone.0079210)
Supplement: Table S1 — Reaction times (in ms) and error rates (in percent) in Experiment 1 as a function of stimulus valence, response goal, order of the response-mapping instructions (congruent task rules first vs. incongruent task rules first), and experiment version (black background vs. white background). Standard deviation in parentheses. (DOCX) [file pone.0079210.s001.docx]

|  |  |  | Turn word on | | Turn word off | |
| --- | --- | --- | --- | --- | --- | --- |
|  |  |  | RT | Error | RT | Error |
| Black version | Congruent first | Positive word | 692 (72) | 1.7 (2.2) | 706 (49) | 4.2 (3.1) |
|  |  | Negative word | 719 (58) | 4.0 (4.5) | 686 (81) | 5.6 (3.8) |
|  | Incongruent first | Positive word | 612 (43) | 5.6 (5.7) | 716 (104) | 5.4 (4.4) |
|  |  | Negative word | 714 (92) | 6.3 (4.1) | 604 (49) | 5.4 (5.1) |
| White version | Congruent first | Positive word | 699 (135) | 7.1 (5.1) | 755 (157) | 7.5 (6.4) |
|  |  | Negative word | 757 (155) | 11.3 (12.0) | 686 (104) | 8.8 (5.8) |
|  | Incongruent first | Positive word | 621 (87) | 4.2 (3.7) | 683 (119) | 3.5 (3.3) |
|  |  | Negative word | 704 (141) | 6.2 (5.0) | 627 (96) | 7.5 (5.7) |
