# Supplementary material for: Affective Compatibility between Stimuli and Response Goals: A Primer for a New Implicit Measure of Attitudes
Source: PLoS One. 2013 Nov 14;8(11):e79210. doi: 10.1371/journal.pone.0079210 (PMC3828340; doi:10.1371/journal.pone.0079210)
Supplement: Table S2 — Reaction times (in ms) and error rates (in percent) in Experiment 2 as a function of stimulus valence, response goal, and order of the response-mapping instructions (congruent task rules first vs. incongruent task rules first). Standard deviation in parentheses. (DOCX) [file pone.0079210.s002.docx]

|  |  | Turn noise on | | Turn noise off | |
| --- | --- | --- | --- | --- | --- |
|  |  | RT | Error | RT | Error |
| Congruent first | Positive word | 795 (124) | 5.1 (6.1) | 748 (108) | 7.7 (6.2) |
|  | Negative word | 767 (116) | 8.0 (6.3) | 800 (117) | 5.9 (5.6) |
| Incongruent first | Positive word | 747 (86) | 6.3 (6.8) | 711 (80) | 1.5 (2.5) |
|  | Negative word | 707 (73) | 4.0 (4.2) | 761 (101) | 7.3 (8.6) |
