# Supplementary material for: Affective Compatibility between Stimuli and Response Goals: A Primer for a New Implicit Measure of Attitudes
Source: PLoS One. 2013 Nov 14;8(11):e79210. doi: 10.1371/journal.pone.0079210 (PMC3828340; doi:10.1371/journal.pone.0079210)
Supplement: Table S3 — Reaction times (in ms) and error rates (in percent) in Experiment 3 as a function of stimulus valence, response goal, and order of the response-mapping instructions (congruent task rules first vs. incongruent task rules first). Standard deviation in parentheses. (DOCX) [file pone.0079210.s003.docx]

|  |  | Turn word on | | Turn word off | |
| --- | --- | --- | --- | --- | --- |
|  |  | RT | Error | RT | Error |
| Congruent first | Positive word | 963 (183) | 10.3 (10.3) | 1067 (259) | 14.7 (12.0) |
|  | Negative word | 1094 (235) | 20.6 (9.8) | 993 (195) | 10.5 (7.5) |
| Incongruent first | Positive word | 824 (126) | 5.5 (5.0) | 1122 (221) | 16.7 (15.2) |
|  | Negative word | 1148 (191) | 16.7 (14.4) | 888 (157) | 11.2 (6.0) |
