# Supplementary material for: Affective Compatibility between Stimuli and Response Goals: A Primer for a New Implicit Measure of Attitudes
Source: PLoS One. 2013 Nov 14;8(11):e79210. doi: 10.1371/journal.pone.0079210 (PMC3828340; doi:10.1371/journal.pone.0079210)
Supplement: Table S4 — Reaction times (in ms) and error rates (in percent) in Experiment 4 as a function of group, exemplar valence, response goal, order of the response-mapping instructions (congruent task rules first vs. incongruent task rules first). Standard deviation in parentheses. (DOCX) [file pone.0079210.s004.docx]

|  |  |  | Raise thumb (top) | | Lower thumb (flop) | |
| --- | --- | --- | --- | --- | --- | --- |
|  |  |  | RT | Error | RT | Error |
| Congruent first | German | Positive exemplar | 940 (174) | 3.4 (4.1) | 963 (141) | 9.1 (9.2) |
|  |  | Negative exemplar | 946 (194) | 7.0 (6.0) | 995 (154) | 10.9 (9.5) |
|  | Foreigner | Positive exemplar | 956 (175) | 10.9 (6.3) | 950 (164) | 9.4 (6.4) |
|  |  | Negative exemplar | 966 (180) | 10.2 (9.4) | 949 (168) | 4.9 (5.7) |
| Incongruent first | German | Positive exemplar | 875 (211) | 3.3 (4.7) | 1131 (210) | 12.2 (10.3) |
|  |  | Negative exemplar | 908 (210) | 5.7 (5.8) | 1157 (196) | 12.8 (10.0) |
|  | Foreigner | Positive exemplar | 1067 (164) | 6.8 (7.8) | 924 (224) | 5.1 (7.7) |
|  |  | Negative exemplar | 1078 (162) | 10.1 (10.4) | 931 (247) | 7.1 (8.1) |
