# Supplementary figures and images for: Affective Compatibility between Stimuli and Response Goals: A Primer for a New Implicit Measure of Attitudes
Source: PLoS One. 2013 Nov 14;8(11):e79210. doi: 10.1371/journal.pone.0079210 (PMC3828340; doi:10.1371/journal.pone.0079210)

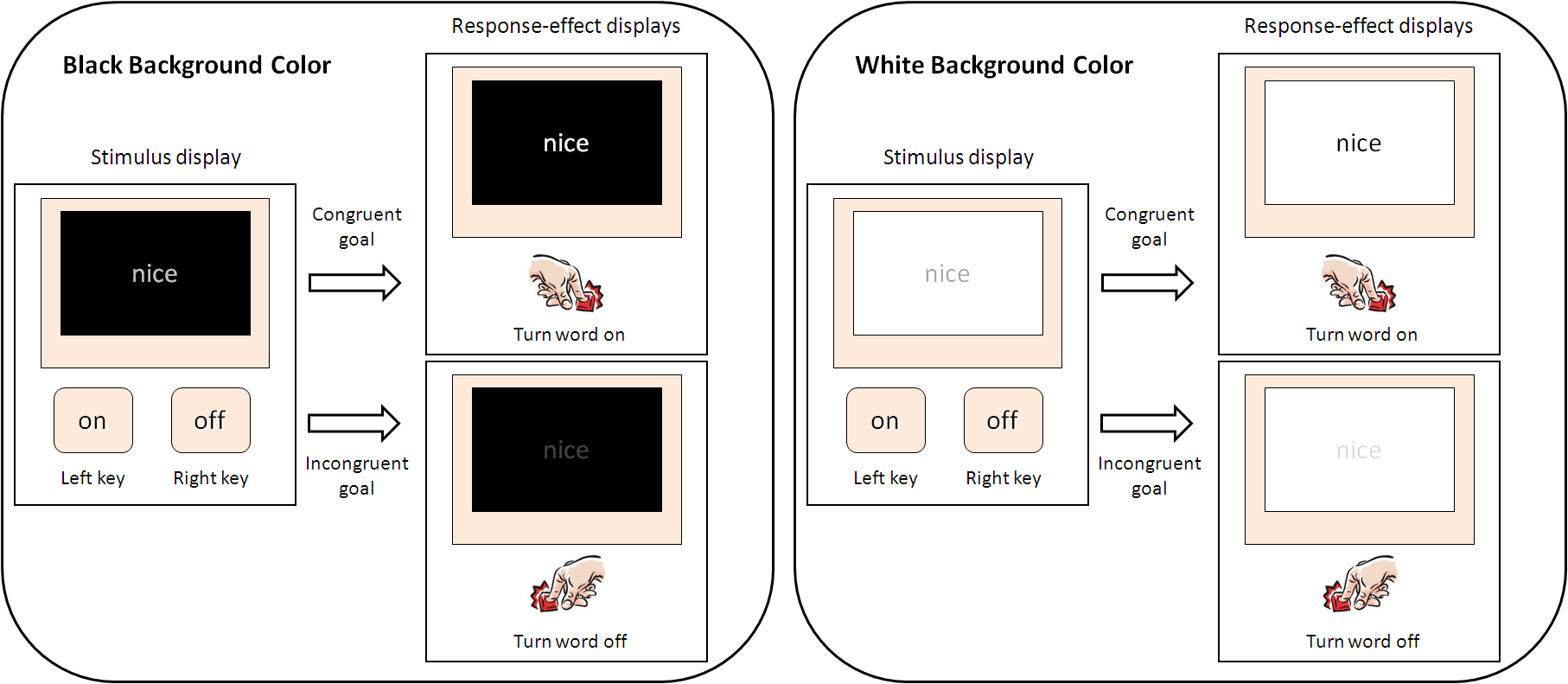

Supplement: Display S1 — Task displays presented in Experiment 1. (TIF) [file pone.0079210.s005.tif]

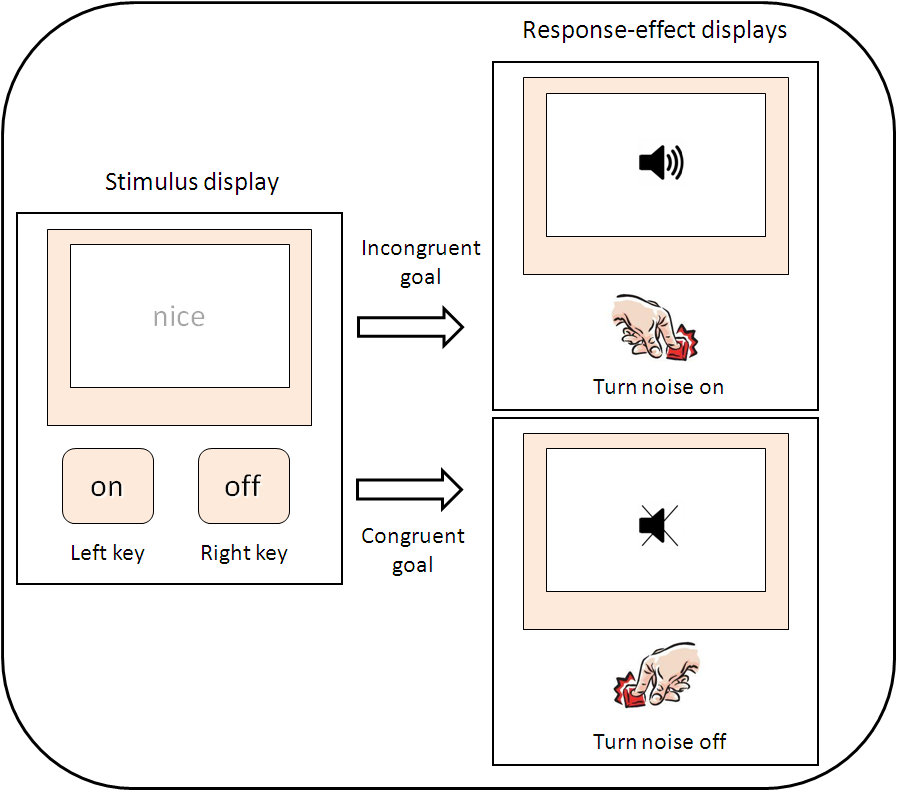

Supplement: Display S2 — Task display presented in Experiment 2. (TIF) [file pone.0079210.s006.tif]

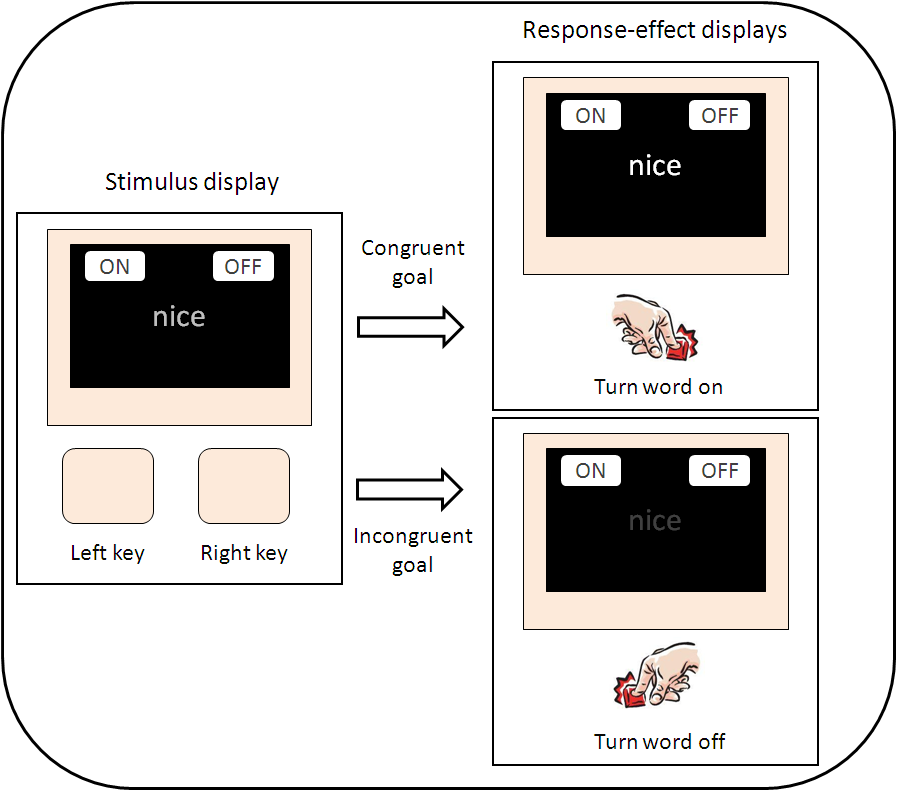

Supplement: Display S3 — Task display presented in Experiment 3. (TIF) [file pone.0079210.s007.tif]

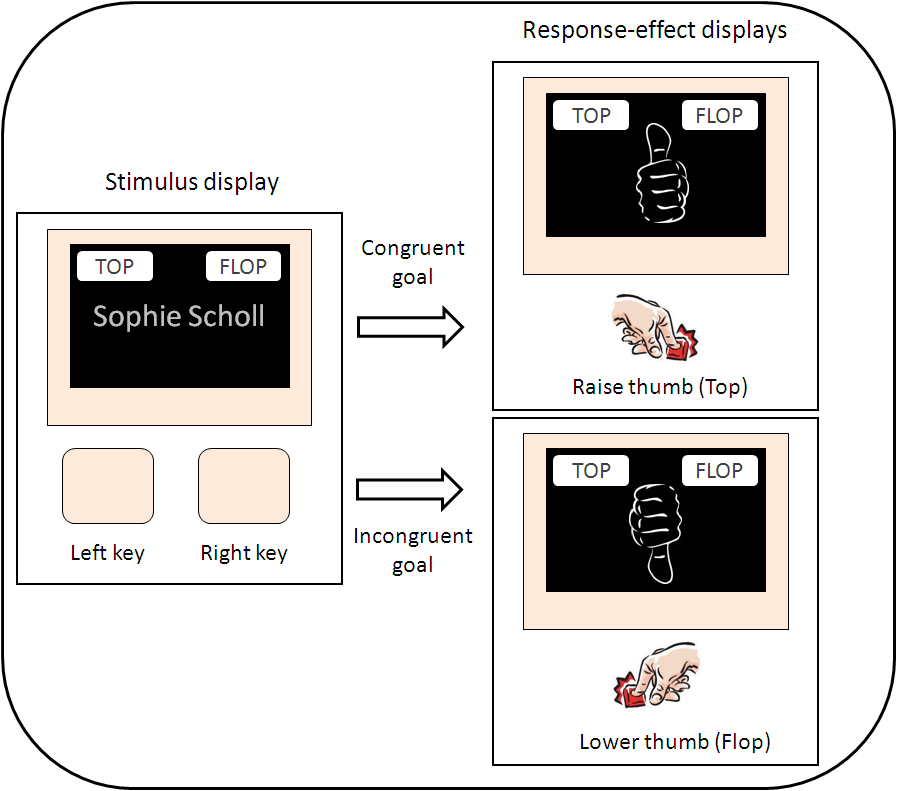

Supplement: Display S4 — Task display presented in Experiment 4. (TIF) [file pone.0079210.s008.tif]
