# Supplementary material for: Affective Compatibility between Stimuli and Response Goals: A Primer for a New Implicit Measure of Attitudes
Source: PLoS One. 2013 Nov 14;8(11):e79210. doi: 10.1371/journal.pone.0079210 (PMC3828340; doi:10.1371/journal.pone.0079210)
Supplement: Stimuli S1 — Positive and negative words presented in Experiments 1 to 3 (English translation in brackets). (DOCX) [file pone.0079210.s009.docx]

**Positive Words**

angenehm [pleasant], besonnen [canny], entspannt [relaxed], findig [resourceful], freimütig [frank], friedlich [peaceful] , gefällig [complaisant], gemütlich [comfortable], gesund [healthy], großmütig [noble], gütig [benevolent], herzlich [cordial], human [human] , korrekt [correct], liebevoll [affectionate], loyal [loyal], musisch [musical], nett [nice], reinlich [tidy] , sachlich [objective], sonnig [sunny], taktvoll [tactful], treu [loyal] , zärtlich [tender]

**Negative Words**

aggressiv [aggressive], anmaßend [presumptuous], böse [evil], boshaft [malicious], brutal [brutal], eitel [vain], furchtbar [dreadful], gefühllos [deadhearted], giftig [noxious], herrisch [bossy], hochnäsig [snobbish], jähzornig [irascible], kaputt [broken], knauserig [penny-pinching], lästig [annoying], launisch [capricious], peinlich [embarrassing], rüpelhaft [rowdily], schlecht [bad], schuldig [guilty], starr [rigid], tödlich [deathly], traurig [sad], zynisch [cynical]
