# Supplementary material for: Affective Compatibility between Stimuli and Response Goals: A Primer for a New Implicit Measure of Attitudes
Source: PLoS One. 2013 Nov 14;8(11):e79210. doi: 10.1371/journal.pone.0079210 (PMC3828340; doi:10.1371/journal.pone.0079210)
Supplement: Stimuli S2 — Target groups and group exemplars presented in Experiment 4. (DOCX) [file pone.0079210.s010.docx]

**Positive German names**

Sophie Scholl (member of the resistance group ‘White Rose’ in Nazi Germany), Romy Schneider (international movie celebrity from Germany), Friedrich Schiller (famous German poet), Ludwig van Beethoven (famous German composer), Günther Jauch (popular showmaster and journalist), Hape Kerkeling (popular comedian and entertainer)

**Negative German names**

Dieter Bohlen (music producer and judge in a talent show), Joseph Goebbels (Minister of Propaganda in Nazi Germany), Beate Zschäpe (member of a Neo-Nazi terror cell in Zwickau), Adolf Eichmann (main organizer of the Holocaust), Desiree Nick (German comedian and “jungle queen” in an entertainment show), Erich Honecker (longtime leader of the former German Democratic Republic)

**Positive foreign names**

Astrid Lindgren (Swedish writer of children’s books, e.g., Pippi Longstocking), Julia Roberts (renowned US actress), Johnny Depp (famous US actor), Mahatma Gandhi (leader of Indian independence movement), Dalai Lama (spiritual leader of the Tibetan people), Nelson Mandela (anti-apartheid activist and former president of South Africa)

**Negative foreign names**

Joseph Stalin (Soviet politician and dictator), George W. Bush (former US-president, 2004-2009), Osama bin Laden (leader of the terrorist organization al-Quaeda), Muammar al-Gaddafi (ruler of Libya and war criminal), Saddam Hussein (president of Iraq and war criminal), Silvio Berlusconi (billionaire and longterm Prime Minister of Italy)
